# Biphasic Activation of Nuclear Factor-Kappa B in Experimental Models of Subarachnoid Hemorrhage *In Vivo* and *In Vitro*

**DOI:** 10.1155/2012/786242

**Published:** 2012-09-23

**Authors:** Wan-Chun You, Wei Li, Zong Zhuang, Yong Tang, Hu-Chen Lu, Xiang-Jun Ji, Wei Shen, Ji-Xin Shi, Meng-Liang Zhou

**Affiliations:** ^1^Department of Neurosurgery, Jinling Hospital, School of Medicine, Nanjing University, 305 East Zhongshan Road, Jiangsu, Nanjing 210002, China; ^2^Department of Neurosurgery, Jinling Hospital, School of Medicine, Southern Medical University (Guangzhou), 305 East Zhongshan Road, Jiangsu, Nanjing 210002, China

## Abstract

It has been proven that nuclear factor-kappa B (NF-**κ**B) is activated as a well-known transcription factor after subarachnoid hemorrhage (SAH). However, the panoramic view of NF-**κ**B activity after SAH remained obscure. Cultured neurons were signed into control group and six hemoglobin- (Hb-) incubated groups. One-hemorrhage rabbit SAH model was produced, and the rabbits were divided randomly into one control group and five SAH groups. NF-**κ**B activity was detected by electrophoretic mobility shift assay (EMSA) and immunohistochemistry. Real-time polymerase chain reaction (PCR) was performed to assess the downstream genes of NF-**κ**B. NeuN immunofluorescence and lactate dehydrogenase (LDH) quantification were used to estimate the neuron injury. Double drastically elevated NF-**κ**B activity peaks were detected in rabbit brains and cultured neurons. The downstream gene expressions showed an accordant phase peaks. NeuN-positive cells decreased significantly in day 3 and day 10 groups. LDH leakage exhibited a significant increase in Hb-incubated groups, but no significant difference was found between the Hb incubated groups. These results suggested that biphasic increasing of NF-**κ**B activity was induced after SAH, and the early NF-**κ**B activity peak indicated the injury role on neurons; however, the late peak might not be involved in the deteriorated effect on neurons.

## 1. Introduction

Aneurysmal subarachnoid hemorrhage (SAH) accounts for 5% of all stroke cases [1], and patients with aneurysmal SAH are at high risk of developing permanent neurological deficits [2]. Based on the failure of clinic treatment focusing on the vasospasm, the researches shifted to brain injury, especially early brain injury (EBI) which is considered the most common cause of disability and mortality after SAH [3, 4]. It has been demonstrated that a number of pathways were implicated in the development of EBI including apoptotic and inflammatory pathways, which have a mutual impact on neurons. 

Nuclear factor-kappa B (NF-*κ*B) is composed of 5 subunits: p65, p50, p52, RelB, and c-Rel, which can form heterodimer or homodimer. The most common heterodimer in central nervous system is p50/p65 [5–8]. NF-*κ*B is a transcription factor regulating numerous genes, such as inflammatory cytokines, apoptotic mediators, growth factors, adhesion molecules, and enzymes, which all are involved in pivotal cellular pathophysiology processes [9–12]. NF-*κ*B is maintained in the cytoplasm in a nonactivated form by combining with an inhibitor subunit, IkappaB (I*κ*B). In response to activating stimuli, I*κ*B is phosphorylated by I*κ*B kinase *β* (IKK*β*), resulting in proteolysis of I*κ*B. Consequently, NF-*κ*B is activated to move into the nucleus, causing mRNA expressions of target genes [13, 14]. NF-*κ*B has been described to contribute to brain injury by inducing the proinflammatory cytokines [15]. Also, on the other hand, NF-*κ*B could promote cell survival by upregulating of antiapoptotic mediators [16]. Many studies showed that NF-*κ*B played a crucial role in self-healing of injured tissues [17–19]. 

Previous studies described the NF-*κ*B activation in neonatal hypoxic-ischemic brain injury [20], ischemic stroke [21], and many other diseases or pathological status. NF-*κ*B was also found to be involved in the pathophysiology of EBI after SAH [22]. As we all know, antiapoptosis and tissue self-healing, which could be induced by NF-*κ*B pathway, must occur in a certain period following SAH. Hence, the full view of NF-*κ*B activation in the brain after SAH should be studied. In this study, we aim to sketch the panorama of NF-*κ*B activation both in a rabbit SAH model (*in vivo*) and in the hemoglobin incubated neurons (*in vitro*).

## 2. Materials and Methods

### 2.1. Animals Preparation

Male New Zealand rabbits (2.0-3.0 Kg) and pregnant mice (16–18 days gestation, used for neuron culture) were purchased from the Animal Center of the Chinese Academy of Sciences (Shanghai, China). The animal procedures were approved by the Animal Care and Use Committee of Nanjing University and in accordance with the Guide for the Care and Use of Laboratory Animals from the National Institutes of Health (NIH). The rabbits and mice were housed under standard conditions of temperature and humidity and a 12 h-light/dark cycle with free access to food and water.

### 2.2. Experimental SAH Model in Rabbits and Experiment Design

One-hemorrhage SAH model was established following the previous operation procedure [23]. Animals were anesthetized with intramuscular ketamine (50 mg/kg) and xylazine (5 mg/kg), and then placed on a heat pad to keep the body temperature at 37°C during experimental procedure. The local hair on the neck was shaved. Subsequently, we sterilized the skin with 75% alcohol. The SAH group was subjected to percutaneous puncture into the cisterna magna. After outflow of 1.5 mL of cerebrospinal fluid (CSF), 1.5 mL autologous nonheparinized fresh arterial blood was injected into the cisterna magna slowly. The rabbits in control group underwent the same operative procedure except for receiving isotonic sodium chloride solution instead of fresh arterial blood. After injection, the rabbits were placed in a head-down position about 30 degrees angle for 30 min and were kept in their normal body temperature. The rabbits were returned to their feeding room after waking up from anesthesia. 

Thirty-six adult male New Zealand rabbits were divided randomly into 6 groups: (1) control group; (2) five SAH groups on day 1, day 3, day 6, day 10, and day 14. Four or eight rabbits per group were used according to special purpose. We set up SAH onset time as day 0 to describe SAH groups. The rabbits were sacrificed under deep anesthesia on 1st, 3rd, 6th, 10th, and 14th days after SAH, respectively. Four rabbits in each group were killed with the previous perfusion method [24] with some modifications. Basically, after the thorax was opened, a cannula was placed in the left ventricle, and then the descending thoracic aorta was clamped using a clip. Later, the right atrium was cut opened, and the rabbits were perfused with 500 mL of physiologic phosphate-buffered saline (PBS) (pH 7.4, 4°C). Finally, the fresh brain cortex was harvested to be placed in frozen liquid nitrogen, and then stored at −80°C for further study. The other four rabbits in control, day 3 and day 10 groups were perfused with 500 mL of 4°C PBS, and followed by 500 mL of 10% formaldehyde under a perfusion pressure of 120 cm H_2_O. Then, half of the whole brain was removed and immersed in 10% formaldehyde for immunohistochemistry. The other half was fixed with 4% paraformaldehyde in PBS (pH 7.4) for 24 h and then was infiltrated with 15% and 25% sucrose solution in turn at 4°C. After that, the brain was stored at −80°C for NeuN immunofluorescent labeling.

### 2.3. Primary Cortical Neuron Culture, Hemoglobin (Hb) Incubated Neuron Injury Model, and Experiment Design

Primary cortical neuron culture was prepared using an established technique [25] with some modifications. Briefly, pregnant mice (16–18 days gestation) were sacrificed by cervical dislocation and were put in 75% alcohol disinfectant for sterilizing. Embryos were removed into precooling PBS using sterile techniques; then, the skull, meninges and vascular tissues were detached from the fetal cortex with the aid of a dissection microscope. Cortex was dissected free and treated by 0.1% trypsin for 5 min at 37°C. Subsequently, the supernatant, which contained trypsin, was discarded. We washed the tissues three times with precooling PBS, and then, triturated them in PBS with fire-polished glass pipettes. The neuron suspension was filtered through a 22 *μ*m-filter into a 15 mL polypropylene conical tube, and then, was sedimented at 1500 g for 5 min at 4°C. The sediment was resuspended in Neurobasal medium (Catalog no. 21103-049, *Life Technologies*) with B27 (Catalog no. 10889-038, *Life Technologies*), penicillin and streptomycin. Finally, neurons were seeded in poly-D-lysine and laminin coated plates and were incubated at 37°C in a 5% CO_2_ atmosphere. 

During the first 10 days *in vitro*, half of the culture medium was replaced every 3 days. The cultures were used on 10th day when the cultures were essentially free of astrocytes [26]. The neurons were treated with Hb at a concentration of 10 *μ*M which was determined from prior studies [27, 28]. After 1, 3, 6, 12, 24 and 48 hours, the neurons were collected for the electrophoretic mobility shift assay (EMSA), real-time polymerase chain reaction (PCR) and lactate dehydrogenase (LDH) detection.

### 2.4. Nuclear Protein Extraction

Nuclear and cytoplasm protein were extracted following the previous studies [24, 27]. 

#### 2.4.1. Cell Nuclear Protein Extraction

Generally, primary cultured neurons were washed twice with PBS and scraped in cold PBS. Then we clustered the neurons and resuspended them with 200 *μ*L ice-cold buffer A, which is composed of 10 mM HEPES (pH 7.9), 2 mM MgCl_2_, 10 mM KCl, 0.1 mM EDTA, 1 mM dithiothreitol (DTT) and 0.5 mM phenylmethylsulfonyl fluoride (PMSF) (all from Sigma-Aldrich Co. LLC. Shanghai, China). The homogenate was incubated on ice for 20 min, and 20 *μ*L of 10% Nonidet P-40 solution was added (Sigma-Aldrich Co. LLC. Shanghai, China). The mixture was stirred vortically for 30 s and spun by centrifugation for 10 min at 5000 g, 4°C. The supernatant was cytoplasmic protein which was discarded. The precipitated nuclear pellet was resuspended in 40 *μ*L buffer B, which contained 20 mM HEPES (pH 7.9), 420 mM NaCl, 1.5 mM MgCl_2_, 0.1 mM EDTA, 1 mM DTT, 0.5 mM PMSF, and 25% (v/v) glycerol. The mixture was incubated on ice for 60 min with intermittent mixing, and it was centrifuged at 13,000 g at 4°C for 15 min. The supernatant containing nuclear proteins was collected and stored at −80°C for EMSA.

#### 2.4.2. Cortex Nuclear Protein Extraction

 Cortex nuclear protein was extracted following a similar procedure as above. Briefly, 100 mg of fresh cortex was homogenized in 0.8 mL of ice-cold buffer A using a fitting pestle. Then 30 *μ*L Nonidet P-40 was added to the system. After centrifuged the mixture, we discarded the supernatant (cytoplasmic fraction) and resuspended the nuclear pellet with 200 *μ*L buffer B. The mixture was centrifuged at 13,000 g at 4°C for 15 min. Finally, the nuclear protein was stored for EMSA. The nuclear protein was quantified with Bradford method before analysis.

### 2.5. EMSA

Commercial kit (Gel Shift Assay System, Promega (Beijing, China) Biotech Co., Ltd) was used to detect NF-*κ*B DNA binding activity. Double-stranded consensus oligonucleotide probe (5′-AGT TGA GGG GAC TTT CCC AGG C-3′) was end-labeled with [*γ*-^32^P] ATP (Free Biotech, Beijing, China) and T4 polynucleotide kinase. Cortex nuclear protein (40 *μ*g in 7 *μ*L) and neuron nuclear protein (2 *μ*g in 7 *μ*L) were incubated at room temperature for 10 min with 2 *μ*L gel shift binding 5x buffer. The mixture was incubated for 20 min with 1 *μ*L ^32^P-labled oligonucleotide probe. Afterwards, the reaction was stopped by adding 1 *μ*L of gel loading buffer and the mixture was resolved on a native 4% polyacrylamide gel in 0.5x TBE (Tris-borate-ethylenediaminetetraacetic acid) buffer. After electrophoresis was conducted at 250 V for 90 min, the bromophenol blue dye ran down three fourths of the gel, then dried the gel with plastic wrap on a gel dryer, and exposed the gel to X-ray film (Fuji Hyperfilm, Tokyo, Japan) at −70°C. Autoradiography and quantification of autoradiographic signal were performed using X-ray film and Un-Scan-It software.

### 2.6. Real-Time PCR

Trizol reagent (Takara Biotechnology Co. Ltd. Dalian, China) was used to extract total RNA from frozen brain cortex and fresh neurons following manufacturer's instructions. Then RNA was reverse-transcribed to cDNA with PrimeScript RT reagent Kit (Takara Biotechnology Co. Ltd. Dalian, China). The primers were synthesized by Invitrogen Life Technologies (Shanghai, China). The primers sequences are shown as below: (1) *rabbit cortex:* IL-1*β*-f TGTCAGTCGTTGTGGCTCT, IL-1*β*-r ACTGTAGTCATCCCAGGTGT; TNF-*α*-f CCACAGCACCCTCAAACCTG, TNF-*α*-r TGCGAGTACACGAAGTAGAGCC; Caspase-3-f ATGCAACAAATGGACCTA, Caspase-3-r GCAAGCCTGAATAATGAA; Bcl-2-f GCTACGAGTGGGATACTGGAGA, Bcl-2-r AGGCTGGAAGGAGAAGATGC; *β*-actin-f AGGCACCAGGGCGTGAT, *β*-actin-r CTCTTGCTCTGGGCCTCGT; (2) *mouse neuron:* IL-1*β*-f GACAGGATGCAGAAGGAGATTACT, IL-1*β*-r TGATCCACATCTGCTGGAAGGT; TNF-*α*-f TCTCATTCCTGCTTGTGGC, TNF-*α*-r CACTTGGTGGTTTGCTACG; Caspase-3-f GACTGGAAAGCCGAAACTC, Caspase-3-r GGCAAGCCATCTCCTCATC; Bcl-2-f TGGGATGCTGGAGATGCG, Bcl-2-r AGGCTGGAAGGAGAAGATGC; *β*-actin-f AGGCACCAGGGCGTGAT, *β*-actin-r CTCAGGCTGGAAGGAGAAGAT. Real-time PCR reactions were performed in 96 well optical PCR plates using applied biosystems step one plus real-time PCR system (Applied Biosystems, Foster City, CA, USA) following the kit protocol. Reactions were conducted in a 20 *μ*L volume of reaction mix with SYBR Premix Ex Taq (2x) 10 *μ*L, forward primer (10 *μ*M) 0.4 *μ*L, reverse primer (10 *μ*M) 0.4 *μ*L, ROX reference dye (50x) 0.4 *μ*L, DNA template 2.0 *μ*L, and dH_2_O 6.8 *μ*L (Takara Biotechnology Co. Ltd. Dalian, China). Thermal cycler protocol: stage 1, 95°C, 30 s for denaturation; stage 2, 95°C, 5 s; 60°C, 30 s; both stage 1 and stage 2 were repeated 40 times, alternatingly. The 2^−ΔΔ*C*_*T*_^ method was used for analyzing the data.

### 2.7. Immunohistochemical Staining

For immunohistochemical staining, NF-*κ*B p65 antibody (NBP1-36209, Novus Biologicals, Littleton, CO, USA; 1 : 100 dilution) was used as primary antibody, and HRP-conjugated goat anti-rabbit IgG (sc-2004, Santa Cruz, CA, USA; 1 : 500 dilution) was used as secondary antibody. The experiment was performed following the manufacturer's protocol. Briefly describing, paraffin embedding brain inferior temporal lobe sections were deparaffinized and then rehydrated in graded concentrations of ethanol. Then, the sections were immersed in citrate buffer (pH 6.0), and heated to 95°C for 40 min to retrieve antigens. After cooling sections at room temperature, horse serum was used to block nonspecific protein binding for 30 min. Primary antibody p65 was used to incubate sections overnight at 4°C, then HRP-conjugated goat anti-rabbit IgG (1 : 500 dilution) was incubated with the section for 30 min, AB enzyme reagent for 30 min. Finally, peroxidase substrate was used for coloration, and hematoxylin was used for counterstain. Digitized images were assembled using Adobe Photoshop software.

### 2.8. NeuN Immunofluorescent Labeling

For immunofluorescent labeling, anti-NeuN antibody (MAB377, Millipore, MA, USA; 1 : 100 dilution) was used as primary antibody, and goat anti-mouse IgG Texas Red conjugate (ab51410, Abcam, MA, USA; 1 : 200 dilution) was used as secondary antibody. The experiment was performed following the manufacturer's protocol. To describe it briefly, frozen inferior temporal lobe sections were used for staining of NeuN. The sections were incubated in 0.1% Triton X-100 for 60 min followed by incubation in 5% fetal bovine serum for 60 min to quench nonspecific binding. Next, the sections were incubated with mouse anti-NeuN overnight at 4°C. Then, Texas Red was used to label NeuN. Digital images were assembled using Adobe Photoshop software.

### 2.9. LDH Quantification

Lactate dehydrogenase (LDH) detection kit (Nanjing Jiancheng Bioengineering Institute, Nanjing, China) was used to quantify LDH release as described [29]. Cell viability was assessed according the ratio of LDH released from damaged cells and total cells. We performed the experiment as previously described [30]. Basically, an aliquot of 250 *μ*L of culture medium was first taken from the neuronal cultures that were grown on a 24-well plate, and then, incubated with the substrate. After collection of the medium, the remaining cells were lysed in 0.9% (w/v) Triton X-100, and LDH content in medium and lysed cells were measured to determine the total LDH content. LDH release from cells was calculated as a percentage of total LDH in each sample.

### 2.10. Statistical Analysis

SPSS 15.0 was used for statistical analysis. Values were presented as means ± SEM. Statistical comparisons between groups were performed using one-way analysis of variance (ANOVA) followed by Tukey's post hoc test. A probability of *P* < 0.05 was considered as statistically significant.

## 3. Results

### 3.1. General Observation

**Figure 1:**
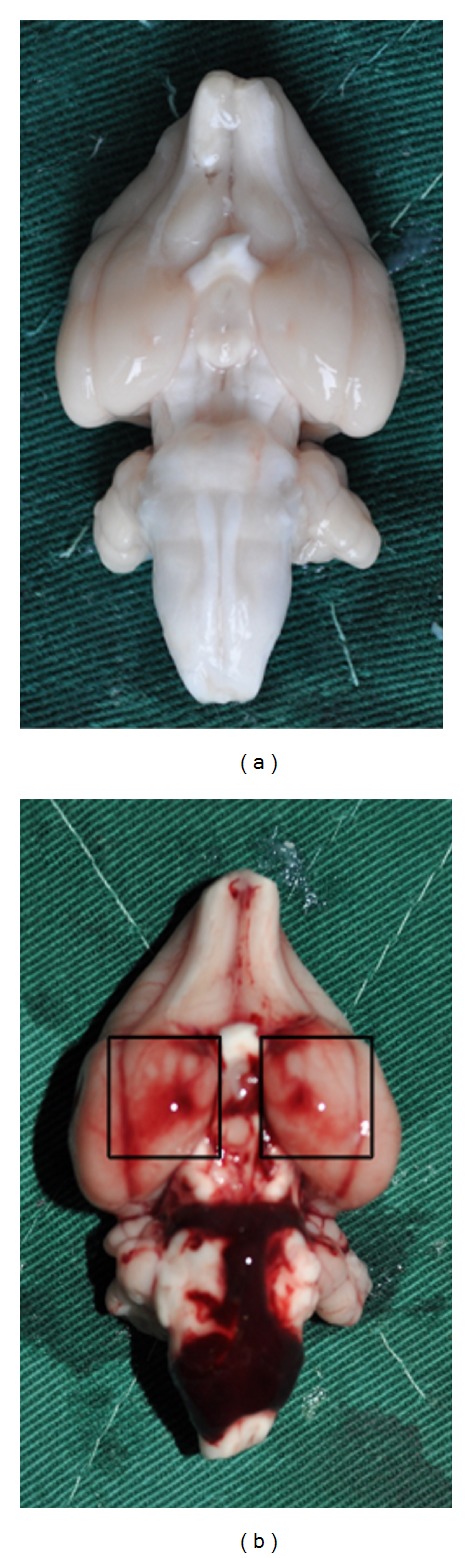
Representative photograph of the brain from control group (a) and day 1 SAH group (b). The brain cortex under the pane is the sample to be detected. It showed that blood clots mainly located in inferior temporal lobe and around the basilar arteries.

There was no death due to SAH in all groups. After SAH, the blood clots could be found on inferior temporal lobe and around the basilar arteries (Figure 1). 

### 3.2. EMSA for NF-*κ*B Activity

**Figure 2:**
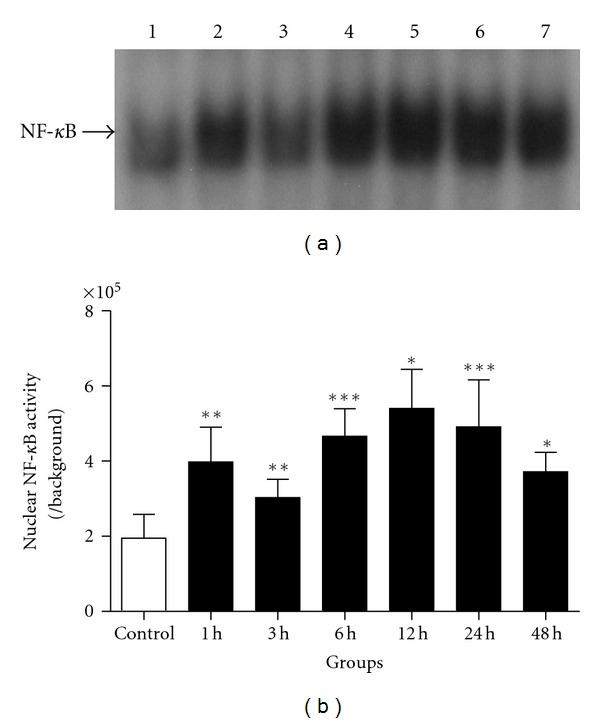
NF-*κ*B activity in Hb-incubated neurons was determined by EMSA in control, 1 h, 3 h, 6 h, 12 h, 24 h, and 48 h groups (lanes 1, 2, 3, 4, 5, 6, and 7 in (a) resp.; *n* = 6 per group). NF-*κ*B activity in cultured neurons was significantly increased after incubated with Hb (*P* < 0.05). The NF-*κ*B activity showed the double peaks in 1 h and 12 h groups, *P < 0.05, **P < 0.01, and ***P < 0.001 versus control group. Bars represent the mean ± SEM.

**Figure 3:**
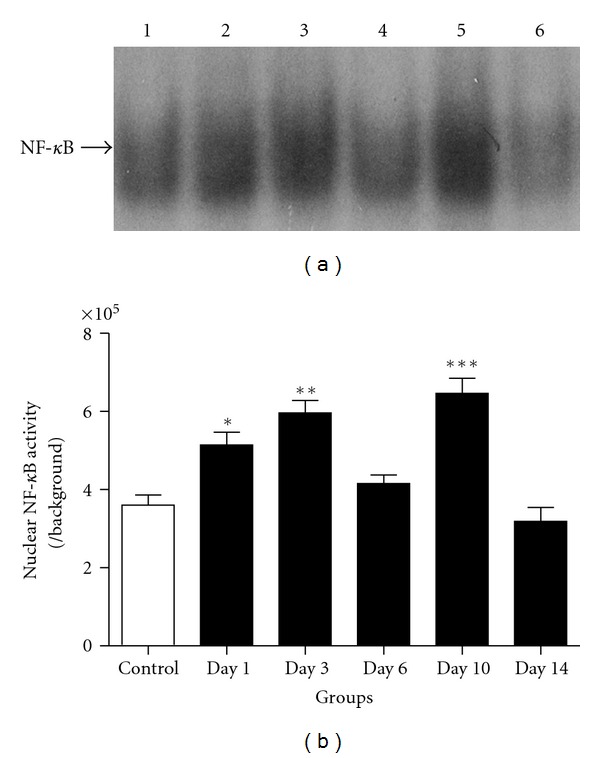
NF-*κ*B activity in brain cortex was detected by EMSA in control, day 1, day 3, day 6, day 10, day 14 groups (lane 1, 2, 3, 4, 5, 6 in (a) resp.; *n* = 4 per group). NF-*κ*B activity showed significant increasing in day 1, day 3 and day 10 group (*P* < 0.05, *P* < 0.01, and *P* < 0.001 resp.). The NF-*κ*B activity showed biphasic peaks after SAH insult, *P < 0.05, **P < 0.01, ***P < 0.001 versus control group. Bars represent the mean ± SEM.

Activated NF-*κ*B in the brain after experimental SAH and cultured neurons was detected by EMSA. The results showed natural biphasic activation of NF-*κ*B both in the animal SAH model and in Hb incubated neurons. *In vitro*, we found that Hb induced the increase of neuronal NF-*κ*B activity dramatically in Hb incubated neurons (*P* < 0.05, versus control group). The increased activity showed double peaks in 1 h and 12 h group, respectively (Figure 2). In the experimental SAH model, NF-*κ*B activity showed double peaks in the brain cortex. The activity significantly increased (*P* < 0.05, versus control group) in day 1, day 3 groups, and in day 10 group (Figure 3). As we saw in Figure 3, NF-*κ*B activity in the other SAH groups did not show significant differences compared with that in control group. 

### 3.3. Immunohistochemistry for p65 Immunoreactivity in the Brain Cortex

**Figure 4:**
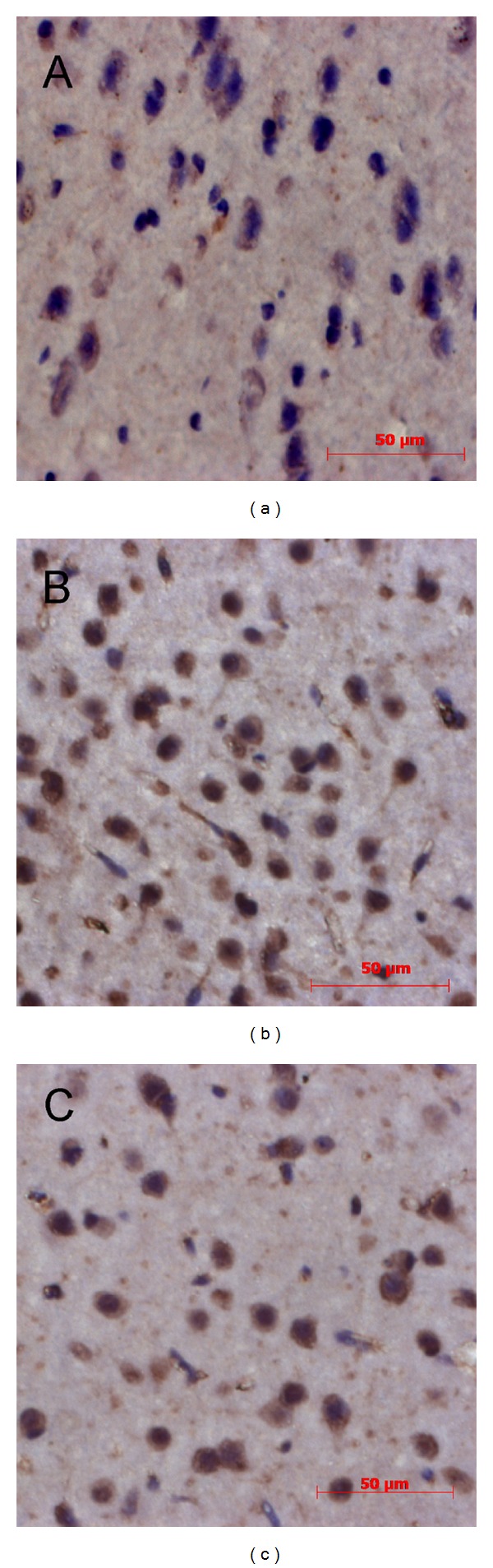
Representative photographs of the NF-*κ*B p65 expression in brain cortex. The subunit of NF-*κ*B p65 was detected by immunohistochemistry in the control (a), day 3 (b), and day 10 groups (c). In the control group, p65 concentrated around the nuclei; while in day 3 and day 10 groups, most of the cellular nuclei were filled with p65. (Scale bar, 50 *μ*m).

Since p65 is the main subunit of NF-*κ*B in the brain, immunohistochemistry for p65 was performed in the brain cortex to assess the location and activity of NF-*κ*B. Immunoreactivity of p65 was observed mainly in the neuronal cytoplasm of the brain in control group (Figure 4(a)). In day 3 and day 10 groups, most of the p65 immunoreactivity was found in nuclei (Figures 4(b) and 4(c)). 

### 3.4. Real-Time PCR for Gene Expressions of IL-1*β*, TNF-*α*, Caspase-3, and Bcl-2

**Figure 5:**
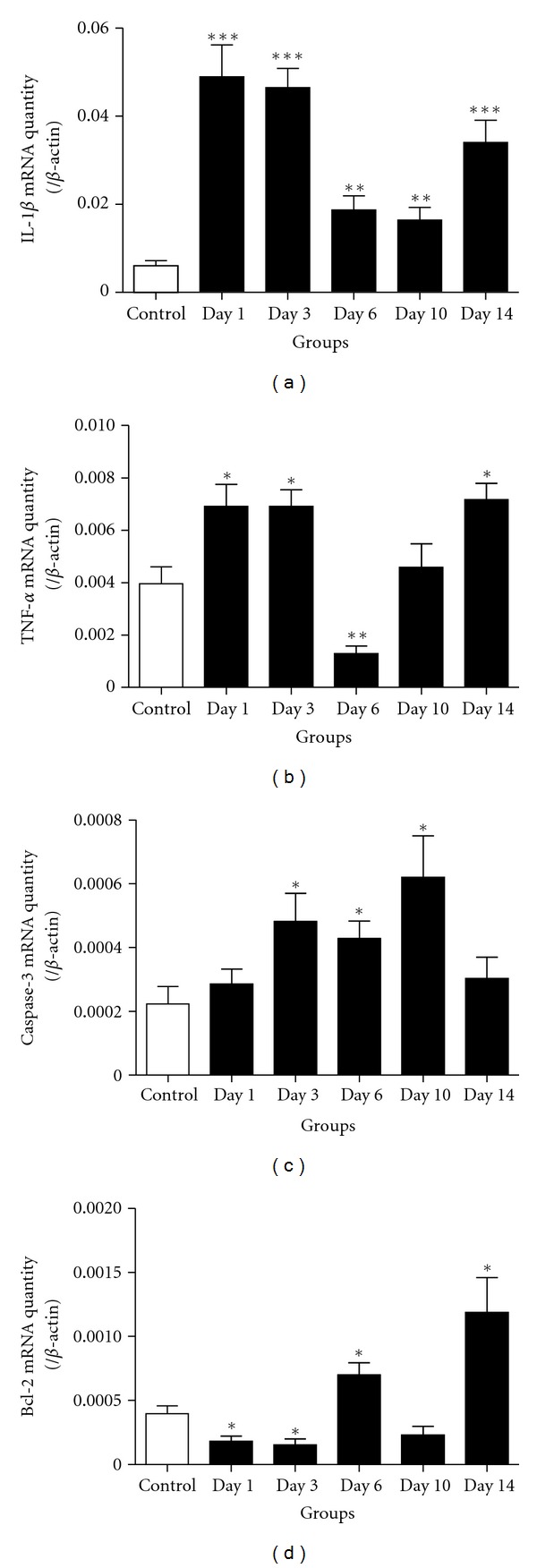
The mRNA levels of NF-*κ*B downstream genes were detected by real-time PCR in brain cortex after SAH. (a) IL-1*β* gene expression was significantly increasing after SAH (*P* < 0.05) and showed a biphasic increasing. Day 1, day 3, and day 14 groups showed the highest expression; (b) TNF-*α* gene showed biphasic expression in day 1, day 3, and day 14 groups; (c) caspase-3 gene significantly increased in day 3, day 6, and day 10 groups (*P* < 0.05), and the expression also showed two peaks on day 3 and day 10; (d) Bcl-2 gene expression was inhibited after SAH. In day 1 and day 3 groups, Bcl-2 was decreased significantly (*P* < 0.05), and in day 6 and day 14 groups, Bcl-2 was obviously increased (*P* < 0.05). *P < 0.05, **P < 0.01, and ***P < 0.001 versus control group. Bars represent the mean ± SEM (*n* = 4, each group).

In experimental SAH model, the gene expression of IL-1*β* increased after SAH and had two peak phases on day 1–3 and day 14 after SAH (Figure 5(a)). The gene expression of another inflammatory mediator TNF-*α* was significantly increased on day 1, day 3, and day 14, which was also, went accordance with the NF-*κ*B activation pattern (Figure 5(b)). Furthermore, the caspase-3 gene expression reached to peak phases in day 3 and day 10 groups, respectively (Figure 5(c)). However, the gene expression of Bcl-2 decreased dramatically in day 1 to day 3 groups, elevated in day 6 and day 14 groups (Figure 5(d)).

**Figure 6:**
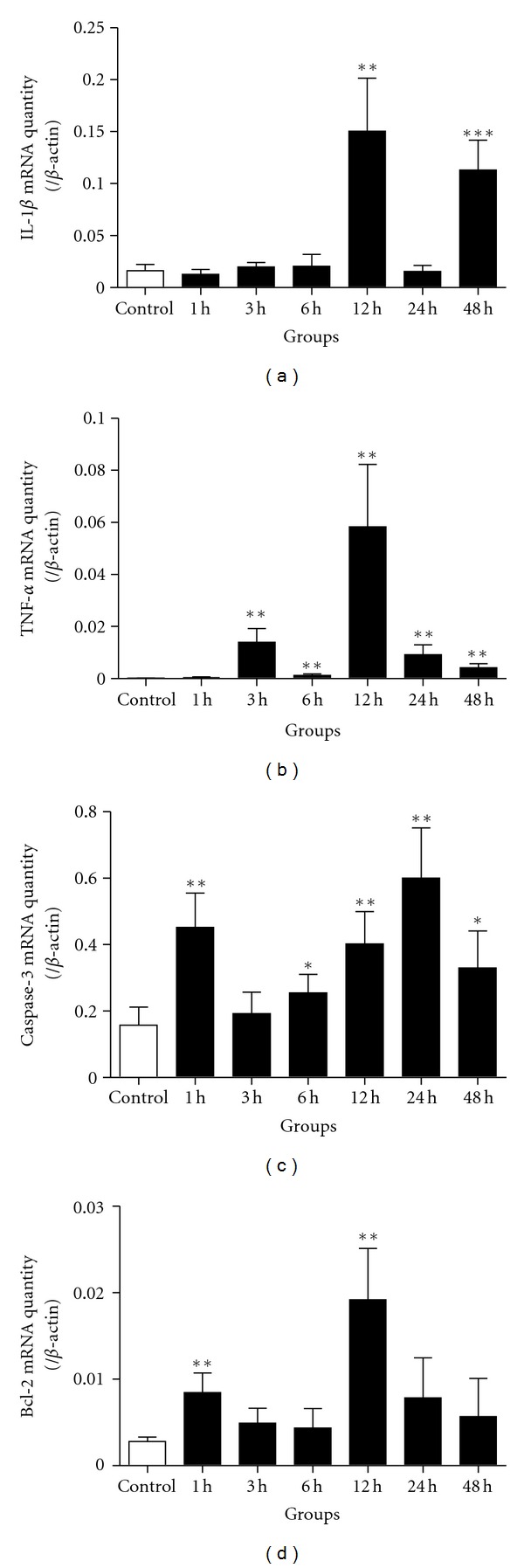
The mRNA levels of NF-*κ*B downstream genes were detected by real-time PCR in Hb-incubated neurons. (a) IL-1*β* gene expression was increased significantly in 12 h and 48 h groups, which showed a biphasic increasing. (b) TNF-*α* gene was dramatically elevated in the groups after 1 hour. There were two TNF-*α* expression peaks in 3 h and 12 h groups. (c) Caspase-3 gene expression also showed two peaks in 1 h and 24 h groups; (d) Bcl-2 gene expression was increased in 1 h and 12 h groups. *P < 0.05, **P < 0.01, and ***P < 0.001 versus control group. Bars represent the mean ± SEM (*n* = 6, each group).

In Hb incubated neuron injury model, the similar tendency chart was found. As showed in Figure 6, the gene expression of IL-1*β* (Figure 6(a)) increased significantly in 12 h and 48 h groups; the TNF-*α* gene expression (Figure 6(b)) was less in the control group and then dramatically elevated in 3 h group. Both the apoptosis-related mediators Bcl-2 (Figure 6(c)) and caspase-3 (Figure 6(d)) showed two peaks in 1 h and 12 h groups.

### 3.5. Quantification of Neuron Deaths with NeuN Immunofluorescent Labeling and LDH Detection

**Figure 7:**
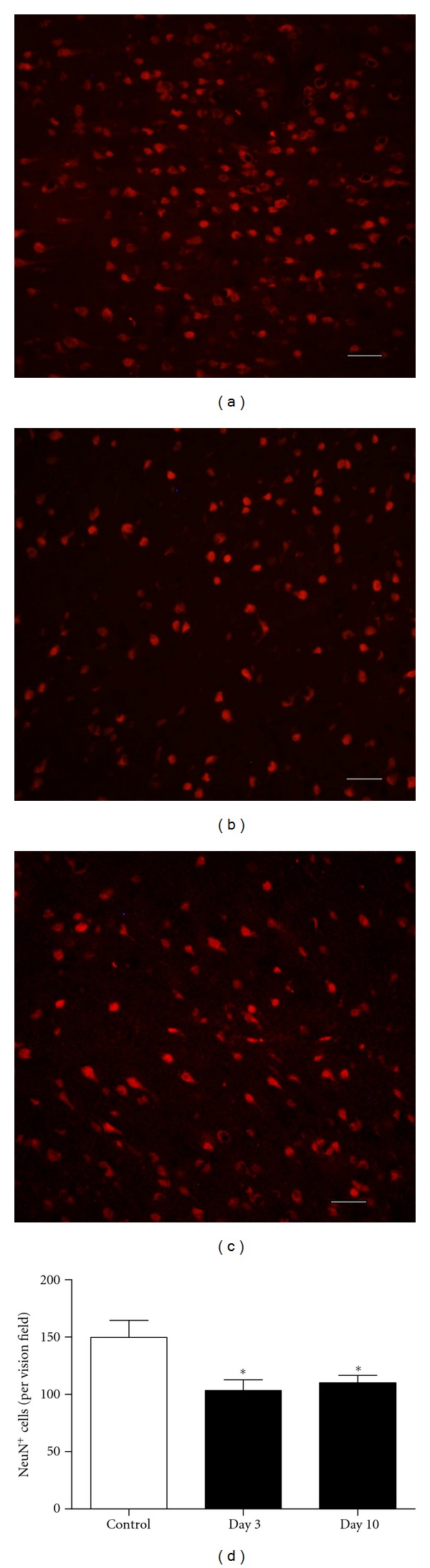
NeuN immunofluorescent labeling was performed for neuron quantification ((a) control group; (b) day 3 SAH group; (c) day 10 SAH group). NeuN-positive cells decreased significantly in day 3 and day 10 groups. There was no statistical difference between two SAH groups. *P < 0.05 versus control group. Bars represent the mean ± SEM (*n* = 4, each group). (Scale bar, 50 *μ*m).

Immunofluorescent labeling was performed to assess the neuron deaths (Figure 7). The results showed more NeuN positive cells in the control group. The NeuN positive cells were significantly decreased in day 3 and day 10 groups versus control group (*P* < 0.05). On the other hand, we found that there was no statistical difference between day 3 and day 10 groups.

**Figure 8:**
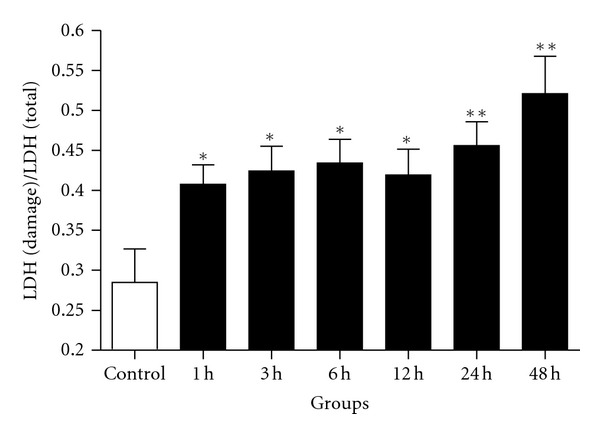
LDH leakage assay was performed for assessing neuron deaths. Cytotoxicity was induced by Hb. Neurons were incubated with 10 *μ*M Hb for 1, 3, 6, 12, 24, and 48 h, respectively. Significant increasing in LDH leakage was found after Hb incubation, *P < 0.05 and **P < 0.01 versus control group. Bars represent the mean ± SEM (*n* = 6, each group).

LDH quantification was used to evaluate the neuron injury after Hb incubation (Figure 8). We assessed the ratio of LDH released from damaged cells and LDH released from total cells. LDH leakage significantly increased in Hb incubation groups compared with the control group. There was no significant difference between any two Hb incubated groups. 

## 4. Discussion

The main findings of this research were as follows. (1) NF-*κ*B was activated after SAH* in vitro* and *in vivo*. The time course of NF-*κ*B showed biphasic activation. (2) The downstream gene transcriptions of NF-*κ*B showed the corresponding changes following the NF-*κ*B activation. (3) The NeuN positive cells in the brain of SAH rabbits showed considerably decrease in the first NF-*κ*B activity peak, and there was no continuous decrease of NeuN positive cells in the late peak. Meanwhile, LDH leakage was always significantly elevated during the early peak in Hb incubated neurons, but there were no increase during the late peak. These findings suggested for the first time that different stages of NF-*κ*B activation may play different roles on neuronal fate in rabbit brain after experimental SAH and Hb incubated neurons.

The rabbit SAH model *in vivo* and Hb induced neuron injury model *in vitro* were used to investigate the activation of NF-*κ*B signaling pathway after SAH in the present study. The detected duration *in vivo* is 14 days after SAH, which covers the early brain injury period and the late brain recovery period after SAH. Our results showed this duration is sufficient for the evaluation of NF-*κ*B activation after SAH. Rabbit one-hemorrhage model, which is proved to be more suitable for brain injury research [31], was produced in the present study. *In vitro*, we cultured the neurons based on previous studies [32, 33]. In Zhang's study, selective inhibition of NF-*κ*B in neuron but not in glia can reduce ischemic brain damage, indicating that NF-*κ*B in neurons may play an important role in the neuron survival. Lara and colleagues indicated that neurons were more efficient in uptake of extravasation of blood such as Hb and heme than astrocytes in intracerebral hemorrhage. The results suggested the neurons were actively involved in brain damage, and Hb was a key agent to induce the pathophysiological process in neurons.

Accumulating evidence demonstrated that NF-*κ*B pathway plays a potential role in the pathophysiological process of various central nervous system diseases such as SAH [24, 34, 35], traumatic brain injury [36], and hypoxic ischemic brain damage [20]. Activation of NF-*κ*B regulates the transcription of inflammatory genes such as TNF-*α*, IL-1*β*, which could activate microglia, could increase the infiltration of inflammatory cells to the brain parenchyma and ultimately could result in brain damage [37–39]. NF-*κ*B promotes cell death in neurons exposed to excitotoxins *in vivo* [40] and *in vitro* [41]. In contrast, Bcl-2, which is regulated by NF-*κ*B, has been proved to promote neuronal regeneration and survival [16]. Furthermore, it has been demonstrated *in vitro* that NF-*κ*B signaling could promote the neuronal stem cell differentiation and asymmetric [17]. These results provided us a new insight of NF-*κ*B for brain repair. In summary, NF-*κ*B is a double-edged sword which has dual roles on neuron survival. 

The biphasic activation of NF-*κ*B was hardly investigated in the previous studies. Nijboer and colleagues [20] described the biphasic activation of NF-*κ*B in hypoxic-ischemic brain damage model for the first time. Interestingly, they found that the different peaks of NF-*κ*B activity played different roles on neuron. In addition, another study detecting Toll-like receptor-4 (TLR4)/NF-*κ*B signaling pathway in the brain after SAH in a rat model showed the biphasic expression of TRL4, which is an upstream receptor of NF-*κ*B [22]. In their article, NF-*κ*B activity was detected in the first two days after SAH in rats. We employed a longer observation time to get the full view of NF-*κ*B activity in our one-hemorrhage rabbit SAH model and detected the NF-*κ*B activity *in vitro *to confirm the results. In the present study, NF-*κ*B activity is significantly elevated in day 1, day 3, and day 10 groups in the rabbit SAH model. Meanwhile, NF-*κ*B showed two significantly increasing activity peaks in 1 h and 12 h groups *in vitro*. The consistent results of biphasic NF-*κ*B activity *in vivo *and* in vitro* may indicate its important role on neuron survival. Combining with the previous findings about the constructive and detrimental effects of NF-*κ*B, we are confident to deduce that biphasic NF-*κ*B activities played conclusive roles on neuronal fate. 

We investigated the mRNA levels of the target genes of NF-*κ*B *in vivo *and* in vitro*. The related gene transcriptions showed significant changes following the NF-*κ*B activity alteration. As the previous studies described, both the pro- and antiapoptotic genes were involved in neuron survival. Caspase-3 was a crucial mediator of pro-apoptosis for neurons [42]. The overexpression of Bcl-2, which is an antiapoptotic mediator, could promote the neuron survival [43]. In present study, we found that caspase-3 gene expression elevated following the peak of NF-*κ*B activation. At the same time, Bcl-2 gene expression altered as NF-*κ*B activity changing which could promote neuron survival. The pro- and antiapoptotic genes showed the same increasing tendency with NF-*κ*B activity, which was confused for the neuronal fate. Therefore, the balance between pro and antiapoptotic genes may influence ultimate neuronal fate. As we know, NF-*κ*B activity in both neurons and glial cells will induce the inflammatory mediators' expressions. The important inflammatory mediators of IL-1*β* and TNF-*α* performed controversial role on the neurons[44]. In our study, two inflammatory mediators elevated significantly to dual phasic peak after insult. NF-*κ*B might modulate the gene transcriptions and protein translations, and finally affected the neuron survival. The exact mechanism calls for further studies. As we known, TNF-*α* could also lead to the phosphorylation of the I*κ*B protein which allows the nuclear translocation of NF-*κ*B and the initiation of its downstream genes transcription [45]. This is a positive feedback loop. We are carrying out more investigation to discover the complex relevance between them.

NeuN stain and LDH detection results suggested that there were significantly decreased neurons and increased LDH leakage during the early NF-*κ*B activity peaks, and no sustained neuron injured evidences were obtained during the late peak. Thus, we inferred that the early NF-*κ*B activity peak promoted neuron death, while the late one did not significantly aggravate neuron damage which might be beneficial for neuron survival. Actually, further research is called to discover the exact mechanism of NF-*κ*B which may determine the neuronal fate. 

## 5. Conclusion

In summary, we found biphasic NF-*κ*B activity after SAH *in vivo *and* in vitro*. Our preliminary data showed that different NF-*κ*B activity peaks performed different roles on neuronal fate. The early peak indicated damage role on neurons survival, and the late peak may not be involved in the deteriorated effect on neurons.
